# On the Slow Diffusion of Point-of-Care Systems in Therapeutic Drug Monitoring

**DOI:** 10.3389/fbioe.2015.00020

**Published:** 2015-02-26

**Authors:** Barbara Sanavio, Silke Krol

**Affiliations:** ^1^IRCCS Fondazione Istituto Neurologico Carlo Besta, Milan, Italy

**Keywords:** therapeutic drug monitoring, point-of-care, anti-epileptic drug, nanomaterials, nanodevices

## Abstract

Recent advancements in point-of-care (PoC) technologies show great transformative promises for personalized preventative and predictive medicine. However, fields like therapeutic drug monitoring (TDM), that first allowed for personalized treatment of patients’ disease, still lag behind in the widespread application of PoC devices for monitoring of patients. Surprisingly, very few applications in commonly monitored drugs, such as anti-epileptics, are paving the way for a PoC approach to patient therapy monitoring compared to other fields like intensive care cardiac markers monitoring, glycemic controls in diabetes, or bench-top hematological parameters analysis at the local drug store. Such delay in the development of portable fast clinically effective drug monitoring devices is in our opinion due more to an inertial drag on the pervasiveness of these new devices into the clinical field than a lack of technical capability. At the same time, some very promising technologies failed in the clinical practice for inadequate understanding of the outcome parameters necessary for a relevant technological breakthrough that has superior clinical performance. We hope, by over-viewing both TDM practice and its yet unmet needs and latest advancement in micro- and nanotechnology applications to PoC clinical devices, to help bridging the two communities, the one exploiting analytical technologies and the one mastering the most advanced techniques, into translating existing and forthcoming technologies in effective devices.

## Introduction

Most of the technological efforts in diagnostic research aims at designing better tools for the diagnosis, treatment, and prognosis of patients’ disease with different strategies, so to provide faster, more sensitive, cheaper, portable, reliable, and less invasive devices (Tüdős et al., 2001; Walt, 2005; Soper et al., 2006; Vyawahare et al., 2010). As the technologies become available, medicine tries to accommodate inter-individual and intra-individual variability into the picture (Hood et al., 2004; Hamburg and Collins, 2010), in an effort to personalize treatment and improve the clinical outcome and the quality of life for the patients (Ginsburg and McCarthy, 2001; Meyer and Ginsburg, 2002; La Thangue and Kerr, 2011). Such efforts may be grouped as biomarker discovery (Rifai et al., 2006; Amur et al., 2008) and monitoring (Phillips et al., 2006; Hamburg and Collins, 2010). This comprehensive approach to identify and measure molecular entities with high-sensitivity (Rusling et al., 2010) provide good, though often surrogate, estimators with good clinical performance in diagnosing or monitoring patient’s disease and treatment (Ginsburg and McCarthy, 2001; Rifai et al., 2006; Kelloff and Sigman, 2012; Pennello, 2013).

The enormous advancements in genomic and proteomic microarrays studies in the latest 15 years have been a driving force toward the development of fast, automatized, high-throughput, multiplexing diagnostic techniques able to reliably process an enormous amount of samples at a time. Usually, the skills required not only for the sophisticated analysis but also especially for the analysis of such large amount of data sets have confined these analyses mainly to large facilities and dedicated centers. Building on the intrinsic high specificity and selectivity of nucleic acid recognition, genetic analysis is the field that experienced the most tremendous innovations, not only on high-throughput ultrasensitive analytical and sequencing techniques but also on miniaturized point-of-care (PoC) devices [see, for example, the very recent review by Kelley et al. (2014)]. Highly sensitive technologies pushed micro- and nanotechnology application toward single cell analysis (Wang and Bodovitz, 2010). In addition, the importance of detecting genetic signatures to correctly diagnose infectious disease, for example, stimulated further extension of miniaturization from the micro- to the nanoscale of DNA analysis for rapid turn-around time (TAT) serotyping of infective agent that with standard techniques (such as culturing the specimen) would require days or weeks.

Biomarker detection research is pursuing clinically significant performance of diagnostic technologies through two main paths. On one side, there is the yet unmet need for ultrasensitive detection of few biomolecules per specimen for early disease diagnosis and surveillance. On the other hand, state-of-the art analytical technologies may have already reached appropriate sensitivity, but have too slow TAT to warrant sufficient clinical outcomes to benefit the patient. Therefore, diagnostic devices with sample-to-answer TAT compatible with a visit at the physician’s office are another promising transformative area of research. Compared to available analytical methodologies, which provide sensitivity with costly, dedicated and sophisticated machinery in centralized labs, new devices are designed to be used at the proximity of the patient, whether this means at the patient’s home, the doctor’s office, the emergency room or in small decentralized, capillary spread laboratories (as drug-stores, for example). Ideally, such new devices would also provide less invasive sampling method and no requirement for sample pretreatment and specialized skills, like the analysis of a blood drop from a fingerprick or saliva from an oral swab. In such a scenario, the target analyte concentration in the biological fluids may fall in a non-analytically challenging range. However, sensitivity issues may still arise as a consequence of the miniaturization and handling of small amount of patient’s specimen containing few analyte molecules.

Few pathological biomarkers are already in the clinical practice (Hartwell et al., 2006), for example, the much discussed PSA, the prostate-specific antigen (Hernández and Thompson, 2004). Clinicians, however, routinely monitor the pharmacodynamic effects of therapies through easily accessible physiological parameters, like blood glucose, glycated hemoglobin (HbA1c), blood pressure, blood cell count, clotting tests, chemico-physical variables like PO_2_, PCO_2_, and electrolytes, and well established panels of metabolic, lipid, liver, renal, and cardiac biochemical indices (Dasgupta and Wahed, 2014).

For many of the easily accessible physiological parameters mentioned earlier, PoC devices are already available both for the physician’s office and the patients (Tüdős et al., 2001; Lippi et al., 2010; Chan et al., 2013) and encompasses different kind of technology (Luppa et al., 2011). Detection of other potentially useful markers falls in analytically challenging concentration ranges, or relies upon detection and read-out technologies that are technically challenging to be re-designed for miniaturization (Cheng et al., 2006; Craighead, 2006; Rusling et al., 2010; Cima, 2011).

In particular, chronic diseases requiring continuous monitoring of therapy (like anti-diabetes and anti-coagulants) have been the first ones for which PoC devices entered the home of the patients (St John and Price, 2014), allowing for timely and constant monitoring of therapy outcome, and improving quality of care over centralized-lab testing (Tüdős et al., 2001; Luppa et al., 2011). A textbook example is the blood glucose meter (Newman and Turner, 2005; Wang, 2008). This extremely portable device seamlessly links the patient to the result of his/her own daily monitoring of therapy. The patient can easily monitor glucose concentration in blood through a fingerprick, and adjust therapy or refer to the physician. In addition, a variety of glucose meter of few centimeters in size is available to the end user for few tens of dollars (plus test strips costs) that devolve the display of results to smartphones (Lillehoj et al., 2013). Finally, the extensive knowledge of the pharmacokinetics (PK) and the pharmacodynamics (PD) of insulin drove the design of automatized closed loop system integrating blood measurements, evaluation, and dosage adjustment and administration in a small, wearable (and soon implantable) lab-on-a-chip (LoC) devices (Hirsch et al., 2008; Cima, 2011; Bandodkar and Wang, 2014).

However, blood glucose concentrations in diabetic patients are in the millimolar range, easily achievable technically compared to the 10^8^ times smaller concentration of most biomarkers (Craighead, 2006; Chin et al., 2012; Chan et al., 2013). The readout is usually an electrochemical determination (most commonly amperometry) of enzyme activity induced by the presence of glucose (Wang, 2008), that can be easily implemented with cheap consumer electronic components. In addition, the frequency and amount of measurements performed daily likely exceed that of any other parameter to be monitored, making the market very appealing (Newman and Turner, 2005). Similarly, PoC clotting tests are also widely used as companion monitoring during anticoagulant therapy (Chin et al., 2012; St John and Price, 2014).

Microfabrication and especially the recent advancement in microfluidics have been the leading protagonists of the development of miniaturized laboratory testing (lab-on-chip) devices, with excellent translation into practice with PoC devices (Tüdős et al., 2001; Vyawahare et al., 2010; Luppa et al., 2011). Recent publications offer detailed reviews of the most recent PoC and LoC devices under development or already available on the market (Toner and Irimia, 2005; Soper et al., 2006; Yager et al., 2008; Mark et al., 2010; Chin et al., 2012; Lisowski and Zarzycki, 2013; Gauglitz, 2014; St John and Price, 2014). As perspicuously detailed by Chin et al. (2012), despite the remarkable advances in the development of single LoC components, *few microfluidic technologies have made the leap to fully functional integrated devices with real clinical value*. Indeed, a plethora of different high-sensitivity techniques emerged in the literature. However, despite the high-performance of the individual parts, the final fully integrated device may not be feasible at all due to poor compatibility of its single components, poor understanding of the end-use settings, and of the critical step in the clinical pipeline. Eventually, they also may demonstrate limited improvement of quality of care compared to the cost reduction or easiness of use.

Trends in the development of PoC devices are different depending on the end-use settings, like high- or low-income countries (Martinez et al., 2010), local clinical laboratory, physician’s office or patient’s home, mobile use, emergency settings (Luppa et al., 2011). The ideal PoC device, however, has to provide rapid TAT sample to answer with no human intervention besides adding the sample and collecting the data. Conventional hematological markers (like the ones mentioned earlier) that are routinely monitored and quantified in clinical laboratory can be evaluated through small bench-top instruments, usually equipped with a series of disposable cartridges for each panel of parameters, which permits the creation of easily accessible diagnostics sites (Pollock et al., 2012; St John and Price, 2014). Automatization and miniaturization, in particular of the fluidic component, drove the development of smaller machinery able to analyze few tens of microliters of patient’s blood (with reduced reagent consumption) (Tarn and Pamme, 2011). These results paved the way to powerful high-throughput, multiplexed, fast platforms, which are very appealing for large facilities and wide genomics/proteomics studies. Quantitative PoC system based on microfluidics and implementing separation and sample pretreatment are gaining increased space in the market (Luppa et al., 2011; Chin et al., 2012). Indeed, additional research goes in the direction of systems able to incorporate or to waive specimen pretreatment, in particular blood separation (Toner and Irimia, 2005; Songjaroen et al., 2012), viscous sample preparation (Ge et al., 2014), and electrophoretic separation (Govindarajan et al., 2011). These principles are implemented in handheld instruments for the detection, for example, of cardiac failure markers that ensure <30 min of TAT directly at the emergency room and improve triage decision making (Chen et al., 2014a), with great clinical impact that largely exceed the added costs (Chin et al., 2012; St John and Price, 2013, 2014).

## Therapeutic Drug Monitoring: A Little Explored Field for PoC?

Parameters like coagulation indices or glycemia are examples of easily accessible surrogate response (pharmacodynamic) biomarkers that monitor the therapeutic effect (Gross, 2001; Touw et al., 2005). For many other therapies, the pharmacodynamic effect is not readily measurable, and large inter-individual variation and narrow therapeutic index make it very difficult to adjust dosage without high risk of toxicity for the patients (Gross, 2001; Buclin et al., 2012). Variability arises in the different absorption and distribution (pharmacokinetic) rates in different patients, that is, the same dosage may translate into different drug concentrations and different pharmacodynamic response to the same drug concentration in different patients (Gross, 2001; Touw et al., 2005; Neef et al., 2008; Buclin et al., 2012). When the population variability is larger than the therapeutic range (that is the difference between the minimum efficacy dose and the lowest dose at which adverse effects arise), monitoring of therapy is crucial.

A long known companion of personalized medicine in these situations is therapeutic drug monitoring (TDM) (Lesko and Schmidt, 2012). The *International Association for Therapeutic Drug Monitoring and Clinical Toxicology* defines TDM (Watson et al., 1997) as “the measurement made in the laboratory of a parameter that, with appropriate interpretation, will directly influence prescribing procedure. Commonly the measurement is in a biological matrix of a prescribed xenobiotic, but it may also be of an endogenous compound prescribed as replacement therapy in an individual who is physiologically or pathologically deficient in that compound.”

Consensus is reached on the rational prescription of TDM, which is – despite recent advancements – a demanding procedure with non-negligible costs for the healthcare systems, both in high- (Schumacher and Barr, 2001; Ghiculescu, 2008; Kang and Lee, 2009; Buclin et al., 2012) and low- (Taur et al., 2013; Nwobodo, 2014) income countries. Guidelines recommend TDM when the following circumstances occur (Gross, 2001; Schumacher and Barr, 2001; Touw et al., 2005; Buclin et al., 2012): (1) when there is a stronger (and ascertainable) relationship between the drug concentration and its therapeutic effect or toxicity, rather than between the administered dose and the effect. (2) If the therapeutic window is small. (3) If no simple accessible parameter is available to evaluate the clinical efficacy. (4) When there is large inter-individual variability in the pharmacokinetic (and sometimes in the pharmacodynamic) parameters. (5) When interaction with co-medications, physiological and pathological changes (intra-individual variations), or compliance issues arise.

Drug monitoring of therapy is helpful in intensive care situations (i.e., aminoglycosides antibiotic, vancomycin, caffeine in neonatal apnea), but is mostly known for chronic therapy such as immunosuppressant, anti-arrhythmic like digoxin, and many anti-epileptic drugs (AED). As their therapeutic index is known to be narrow, administration of these drugs is often accompanied with TDM. The list of most commonly monitored drugs (Gross, 2001; Schumacher and Barr, 2001; Dasgupta and Wahed, 2014) that meet the aforementioned criteria include – but is not limited to – antiretrovirals (Acosta et al., 2002), carbamazepine, valproic acid, phenobarbital phenytoin and lithium among anticonvulsants (Eadie, 2002), aminoglycosides and vancomycin among antibiotics, cardiac drugs like digoxin (Valdes et al., 1998), immunosuppressant as cyclosporine, tacrolimus, mycophenolate mofetil (Johnston and Holt, 2001); anti-cancer drug as methotrexate, bronchodilators as theophylline and caffeine, tricyclic antidepressant among psychiatric drugs (Hiemke et al., 2011).

Drug monitoring helps to identify the onset response at the beginning of therapy, and to adjust the dose in critical patient (with renal, hepatic, or cardiac impairment, etc.). Then, it can be performed at wider time interval to assess efficacy of the therapy, to build a reference range for the patient under evaluation (Patsalos et al., 2008). Moreover, intra-individual variation may occur in the life of a patient due to the appearance of other conditions over time (additional therapy, hepatic/renal/cardiac dysfunction, pregnancy) that alter the metabolism of the drug and require dosage adjustment (Patsalos and Perucca, 2003; Tomson, 2005; Adab, 2006). TDM is an exceptional tool to better understand why patients do not respond satisfactorily to a particular dose, and to assess and monitor compliance and ultimately to study the variation in PK that occurs in different individuals and the factors involved (Muller and Milton, 2012).

Anti-epileptic drugs are a very representative case of TDM application to clinical practice (Neels et al., 2004; Patsalos et al., 2008; Krasowski and McMillin, 2014). These drugs treat and prevent the clinical manifestations, like seizures, only if an effective drug concentration is reached, and present serious side effects when reaching toxic concentration. Most of these drugs have narrow therapeutic index and large inter-individual variability (Eadie, 2002; Johannessen et al., 2003; Patsalos et al., 2008). Historically, the old generation anti epileptic drug phenytoin was one of the first drug for which a TDM test was developed (1960) (Eadie, 2002; Patsalos and Berry, 2013). Phenytoin presents both a non-linear PK (that is, there is a non-linear relation between the administered dose and the blood drug concentration) and high-inter-individual variability. Other old generation anticonvulsants like carbamazepine, valproic acid, ethosuximide, and phenobarbital are still used and monitored (Jannuzzi et al., 2000; Eadie, 2002; Patsalos et al., 2008; Krasowski and McMillin, 2014). Second generation AEDs were marketed as safer because of a wider therapeutic index, though practice revealed that achieving proper dosage is challenging and TDM could be useful (Perucca, 2000; Johannessen et al., 2003; Striano et al., 2008; Krasowski, 2010; de Leon et al., 2013). Being epilepsy a chronic disease that affect almost 3% of the population worldwide, for which TDM was firstly introduced almost half a century ago, one would expect a rather diffused availability of PoC devices for home-based therapy management.

Many recent analyses tried to assess the cost–benefit for TDM of different classes of drugs, and only a few were able to clearly assess the superior clinical performance – in terms of cost-effectiveness – of routine TDM (Eadie, 2002; Touw et al., 2005; Tomson et al., 2007; Salih et al., 2013). AEDs TDM is usually perceived as cost-effective, because of the high risks associated with improper dosage and the successes achieved in managing therapy with the proper interpretation of TDM analysis (Glauser and Pippenger, 2000; Buclin et al., 2012). In addition, for AEDs and many other drugs, TDM studies would provide invaluable information on the PK of drugs in subpopulation not well represented in the pharmacokinetic studies during trials, such as gender (Morrell, 1996; Tomson, 2005), pregnancy and newborns (Adab, 2006), children (Momper and Wagner, 2013), and elderly people (Steinman et al., 2011). While the contribution of TDM to the effective management of therapy is substantiated by practice, properly designed studies are missing, or evaluate too different clinical outcome variables that prevent proper comparison (Touw et al., 2005; Tomson et al., 2007; Salih et al., 2013). As health-care systems aim at providing services at reduced cost whenever possible, rationalization of costly TDM analysis prescription is everywhere highly recommended (Touw et al., 2005; Ghiculescu, 2008). Unfortunately, the lack of consensus on a clear-cut quantitative analysis in favor of distributed, decentralized TDM procedures is slowing down the investment and applications of PoC system in the field.

## Traditional TDM Pipeline

There is heterogeneity in how TDM is accomplished in different centers (Gross, 2001; Schumacher and Barr, 2001; Neef et al., 2008; Kang and Lee, 2009). Besides different analytical methodologies, and different specimen analyzed, there are differences in the kind of information that is provided to the treating physician. For the value to be informative, drug measurements need to be accompanied by proper detailed information about dosing schedule, last dose administered timing, and sample timing (Aronson and Hardman, 1992). Anyhow rarely, TDM service may provide the drug concentration value *per se*, but more often it is accompanied at least by a reference range (validated for the sample and technique employed) and with suggestion for clinical interpretation to be translated in therapeutic management. This reference range is – as the name suggests – just a reference interval, statistically defined, that encompasses the spread of the concentrations in a population of patients addressed during clinical studies. It may change for different therapeutic uses of the same drug, and exact values of the boundary limits may change depending on the analytical technique involved. The role of clinical pharmacologists is therefore gaining increasing interest as novel theoretical approaches to PK and PD studies allow for highly predictive model to estimate proper dosage adjustment from the analysis of the patients’ drug concentrations. Other critical details are co-medications and demographics, like age, gender, and eventually ethnicity [as pharmacogenomics studies distinguished specific, in distinct subpopulations, in metabolic enzymes responsible for altered metabolism and toxicity risks of certain drugs (Gurwitz et al., 2003; Anderson, 2008)].

Unfortunately, as Phase II and Phase III of drug development clinical trials require dose tolerance and dose response exploration only, PK investigations are often a secondary objective (Touw et al., 2005; Neef et al., 2008). Most of the drugs reaches Phase IV (the post-marketing phase) with very little information on the PK and PD behavior of the drug, and rarely with estimation on the appropriateness of accompanying TDM, which is left to the responsibility of practitioners (Gross, 2001; Neef et al., 2008; Muller and Milton, 2012; Momper and Wagner, 2013). In consequence, decentralization of the TDM analysis have been perceived as very critical and potentially detrimental to the control of patient’s therapy, as many general practitioner do not have the expertise to provide interpretation of the analytical results. Moreover, for most drugs, the need for TDM in proper management of therapy was discovered only in clinical practice.

However, in principle, having the PK and PD data available for the monitored drug, current computational techniques would allow full integration of these calculations in portable devices to provide a proper reference range delivered with the analytical answer, and would allow for fast results during the visit at the physician office (for example), and for direct communication of the results to the centralized lab for continuous therapy surveillance. These pieces of information help, together with the clinical and dosage history of the patient and the knowledge of the PK and PD profiles of the drug, the clinical interpretation of the measured value and inform proper changes to therapy and therefore improving the patient’s benefit and minimizing hospitalization and hospital personnel costs.

Many groups call for mandatory TDM in drug development clinical trials (Neef et al., 2008; Muller and Milton, 2012), which require the concurrent development of an *in vitro* diagnostic test for the drug (Pirmohamed, 2010; Shimazawa and Ikeda, 2015). Concentration-Controlled Randomized Clinical Trials (CCRCT) design was suggested more than 20 years ago (Sanathanan and Peck, 1991), because of the improved sample size efficiency, but it is still very little implemented (Lledó-García et al., 2009; Momper and Wagner, 2013). Advancement in PK–PD modeling allow for prediction of the average response profile to a given dosage and evaluation of a therapeutic margin; for identification of susceptibility factors that alter the therapeutic/toxic response, and for quantification of variability response, that can account also for random population variability (Lledó-García et al., 2009; Muller and Milton, 2012; Momper and Wagner, 2013). Establishment of proper PK–PD characterization during drug development generates detailed knowledge of PK–PD parameters that constitute solid reference for translation of dosage adjustment (or for subsequent clinical trials) in the targeted patient’s population and, more importantly, in subpopulations overly under-represented in drug development (Walson, 1998), like pregnancy (Ke et al., 2014), infants (Koren, 1997; Momper and Wagner, 2013), and elderlies (Battino et al., 1995; Perucca, 2005, 2006; Steinman et al., 2011; Etwel et al., 2014).

Besides the burden of additional blood sampling to the enrolled patients, the main hurdle to TDM inclusion in drug development and investigational trials (and practice) is cost (Neef et al., 2008; Shimazawa and Ikeda, 2015). Indeed, incorporation of TDM in drug development requires additional investments from the industry in the development of a robust, validated bioanalytical assay. In addition, TDM is somehow also perceived by companies as a potential barrier for the subsequent effortless adoption of the drug in the clinical practice (Neef et al., 2008; Shimazawa and Ikeda, 2015). Both the increase in costs for drug developments along with a lack of awareness of the regulatory authorities and hence the obligation for companying TDM during drug development and approval led to the fact that little investment and research effort is made in the field, even so the new developments in terms of nanotechnology for sensor techniques are promising.

The main points where improvements need to be made and can be gained with nanotechnologies are to increase the sensitivity in order to work with smaller sample volumes allowing for a repeated sampling or even more non-invasive sampling such as tears or sweat. Smaller volumes also mean that the time of reaction with the sensor system decreases and hence the time-to-result.

The most commonly employed technologies for TDM so far have been extensively revised for the different classes of drugs [see, for example, Dasgupta and Wahed (2014)]. Usually, drug concentration is monitored from a plasma sample, though whole blood could also be analyzed (Aronson and Hardman, 1992; Reynolds and Aronson, 1993). Sample treatments extract the total drug available in plasma that is the sum of the protein-bound and free drug in circulation. Depending on the assay chosen, the analytical protocol has to be validated at each step from the pre-analytical phases to result communication and storage. The pre-analytical phase includes patient preparation, sample collection, sample treatment, and is often unique to the drug to be analyzed. For example, lithium therapy monitoring cannot be performed on sample collected in standard lithium heparin blood tubes, and colorimetric tests may suffer the interference of a poorly drawn blood sample (from hemoglobin interference) (Aronson and Hardman, 1992; Reynolds and Aronson, 1993; Dasgupta and Wahed, 2014).

Analytical separation techniques are widely established methodologies for TDM. High-performance liquid chromatography (HPLC) (Taylor, 2005), capillary electrophoresis (EC) (Kataoka et al., 1998; Pucci and Raggi, 2005), liquid or gas chromatography (LC or GC) coupled with UV spectroscopy (Serralheiro et al., 2013), or mass spectrometry (MS or eventually tandem MS, MS/MS) (Maurer and Peters, 2005) have been extensively applied to different varieties of pharmaceutical and abuse drugs with excellent performances (Brandhorst et al., 2012), with optimized protocols for the simultaneous separation and analysis of multiple drugs at a time, such as in the case of co-medication therapy, or simply, when analysis of a drug and its metabolite(s) is necessary.

Appropriate sample pretreatment is required. It involves solvent or solid-phase extraction (Bugamelli et al., 2002; Vermeij and Edelbroek, 2007; Tonic-Ribarska et al., 2012) of the analyte from the biological matrix, though some publications report direct injection of plasma into an online separation/analysis system (HPLC-MS) (Martinavarro-Dominguez et al., 2002). These techniques rely upon sophisticated and expensive equipment that has to be operated by technically skilled personnel and are usually more common in large clinical research laboratory.

**Figure 1:**
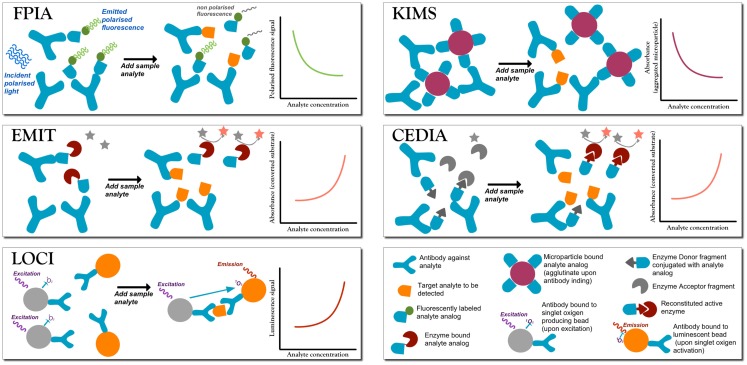
**A summary of the most commonly employed homogeneous competitive assays in TDM (Touw et al., 2005; Dasgupta and Wahed, 2014)**. In fluorescence polarization immunoassay (FPIA) after incubation, the fluorescence polarization signal is measured without separation of bound from free labels. Free labeled analyte analog molecules are added to the sample, and it has a different Brownian motion than when the label is bound to a large antibody (Ab). When the analyte is present, there is competition for the binding to the Ab. If the labeled analyte is bound to the Ab molecule then the signal is generated, while when the labeled antigen is free in solution no signal is produced. Therefore, signal intensity is inversely proportional to the analyte concentration. Enzyme multiplied immunoassay technique (EMIT). Free analyte analog molecules labeled with an enzyme, e.g., glucose-6-phosphate dehydrogenase enzyme, are added to the test solutions to compete to the analyte to be tested. The active enzyme reduces NAD (no signal) to NADH (absorbs at 340), so that absorbance is monitored at 340 nm. When labeled analyte binds to the Ab, the enzyme becomes inactive, and so the signal is generated by the free label, and signal intensity is directly proportional to the analyte concentration. Luminescent oxygen channeling immunoassay (LOCI). The reaction mixture is irradiated to generate singlet oxygen species in microbeads coupled to the analyte. When bound to the respective Ab molecule, also coupled to another kind of bead, the analyte reacts with singlet oxygen and chemiluminescence signals are generated proportionally to the concentration of the analyte–Ab complex. Kinetic interaction of microparticle in solution (KIMS) and the conceptually similar particle enhanced turbidimetric inhibition immunoassay (PETINIA). In the absence of the analyte, free antibodies bind to drug microparticles conjugates to form aggregates that absorb in the visible range. Absorbance (or turbidimetry) is monitored and in presence of the analyte the Ab binds to the free analyte preventing microparticle aggregation; a reduction in absorbance is observed (signal is inversely proportional to analyte concentration). Cloned enzyme donor immunoassay (CEDIA). An enzyme (like beta-galactosidase) is genetically engineered into two inactive fragments: a small one called enzyme donor (ED) conjugated with the drug analog, and a larger fragment enzyme acceptor (EA): when the two fragments associate, the full enzyme converts a substrate into a cleaved colored product. If drug analyte molecules are present, they will compete with the ED-labeled drug in solution for the limited Ab sites, so that free ED-labeled drug analog will bind to EA generating a colorimetric signal directly proportional to the amount of analyte.

In large laboratory hospitals, immunoassays with different read-out technologies, ranging from nephelometry, chemiluminescence, to colorimetry and fluorescence (see Figure 1 for a survey of these techniques), are available for a panel of drugs in multi-well plate assays with ready-made proprietary reagents compatible with automatized commercial system already in use for other analyses. These commercial systems often include automatized sample pretreatment. Protocols may differ among centers, and each center performs a proper analytical validation of the methodological protocol and the entire process, as guidelines require ensuring accurate and robust analyses (Shipkova et al., 2014). Some immunoassays fall short of simultaneous determination of a drug and its active metabolite (Mikel et al., 2012), or in the presence of co-medications that may cross react with the antibody used for the assay. Different commercial assays present different (or none) cross-reactivity patterns, for many of which the literature offers details (Kang et al., 2011; Brandhorst et al., 2012; Shipkova et al., 2014), though the expertise of the clinical laboratory is pivotal in the recognition of interfering factors in abnormal analyses.

Compared to sophisticated techniques, large-scale technique like GC-MS, HPLC-UV, or HPLC-MS/MS (and variations) requiring sample volumes of some milliliters, immunoassays are easily and readily miniaturized by employing nanostructured surface sensors or micro and nanoparticles (NP), and microfluidic versions of these analytical devices are already available for research purposes. However, as most of the immunoassays rely upon plasma samples, pre-treatment is an issue, and the lack of integrated sample treatment in a miniaturized device is going to impede its usefulness as a PoC technology. Typically, therapeutic drug concentration are measured in plasma, but also whole blood or saliva are tested (Chiappin et al., 2007); other biological matrices such as hairs, tears, meconium, stool, urine, cerebrospinal fluid (Caplan and Jenkins, 2008; Neef et al., 2008; Buclin et al., 2012), and sweat are rarely used in clinical TDM routine, but are often explored in drug abuse and patient’s compliance investigations (George and Braithwaite, 2002).

There are special cases, like neonates, children, and adolescents (MacLeod, 2010), which require more frequent monitoring from the onset to the stabilization of therapy. Indeed, pharmacokinetic parameters present wide inter-individual variability at the neonatal and infancy stage, and undergo rapid maturational changes that make safe and effective dosing a burdensome challenge (Perucca, 2005; MacLeod, 2010). Later in development, patient’s growth requires therapy adjustments that must be individualized, as growth rates are different among children population. Cheaper, sensitive, miniaturized devices able to shift the monitoring at the physician office or at the patient’s home would allow for meticulous and less stressful and invasive time sampling, e.g., at the manifestation of toxic symptoms, or saving patient’s and skilled personnel time. Such an implementation would improve the clinical performance of the monitoring by improving not only cost containment but also, most importantly, patient’s quality of life.

## Toward PoC Monitoring of AEDs

To the best of our knowledge, very little of PoC technologies have been translated to therapeutic AED monitoring. Development of novel, cheap, and eventually PoC devices seems to be trapped into a paradox. Despite the transformative potential on therapy management, investors seem to be not significantly attracted by the development of novel devices, because of the lack of clear meta-analysis and trials on the cost-effectiveness and cost-utility analysis of TDM practice (Billings, 2006). TDM trials do not receive adequate funding as it is not yet mandatory for industries to include TDM in drug development and government may prefer not to fund with public grants research on industry’s marketable products. In addition, despite the fact that the new Horizon 2020 is focused on translation of research to industrial products, TDM was not identified as a potentially under-financed field. At the same time, the lack of a simple, easy-to-use, and cheap device that reproducibly measures drugs in patient-friendly matrices slows down the collection of useful PK and PD data able to improve TDM practice (Neef et al., 2008). A promising recent study is employing the bio-nanochip device (Jokerst and McDevitt, 2010; Du et al., 2011) for saliva monitoring of phenobarbital and phenytoin through a micro-bead assisted immunoassay, ready to be employed into a clinical trials for TDM in epileptic children. The chip comprises a programmable chemical processors and assay AEDs with a bead-based immunoassay. It is the first pilot study of the adaptation of an existing fully integrated (miniaturized) PoC device to AEDs TDM (Fitzgerald, 2014).

However, some centers have simply and elegantly circumvented the need for hospital visit in TDM, especially in monitoring of pediatric patients, through collection of dried blood spot (DBS) (Edelbroek et al., 2009; Wilhelm et al., 2014). DBS was introduced by Guthrie and Susi (1963) for low invasive neonatal screening of metabolic disorders. Paper substrates and sampling guidelines are well characterized in this respect, as rigid quality standard requirements are present, thus translation into TDM practice was facilitated, though no established specific guidelines are presents for TDM. The patient, the caregiver, or the personnel of small laboratories collect a drop of peripheral blood on a marked paper card that can be mailed back to the reference hospital, where the spot is treated so to extract the drug to be analyzed (Edelbroek et al., 2009; Demirev, 2013; Shah et al., 2013; Ostler et al., 2014). The possibilities of saliva as a vehicle to carry out non-invasive monitoring (Aps and Martens, 2005; Chiappin et al., 2007; Schipper et al., 2007; Malamud, 2011) has been long explored and identified some AEDs for which the fluid is a suitable medium for their measurement (Liu and Delgado, 1999; Jones et al., 2005; Patsalos and Berry, 2013), particularly in children or if monitoring needs to be done frequently (Liu and Delgado, 1999; Tennison et al., 2004; Patsalos and Berry, 2013).

Both strategies (saliva and DBS) open the prospect of strategically timed self-collection of the fluid by the patient in his or her home, providing for direct assessment of drug concentration as seizures, intermittent symptoms or side effects occur. More over, they offer patient-friendly access to additional sampling for PK and PD studies during trials (Chee et al., 1993; Gorodischer et al., 1997; Kong et al., 2014). Although mailing the samples back to the hospital does not shorten the time necessary to get the TDM evaluation (Jones et al., 2005), the procedure allows for the monitoring of the patient at the exact time of the discomfort symptoms. This provides useful information to proper therapy individualizations ensuring better management of adverse effects, which are the major cause of patient’s reduced compliance. Posting of samples to the laboratory (Tennison et al., 2004; Jones et al., 2005; Wilhelm et al., 2014) for subsequent analysis translates into saved costs both as personnel time and as improved quality of life for the patient. If the outcome of therapy management is measured solely as, for example, the patient’s seizure-free interval, probably the differences between centralized and PoC testing performance are not going to be sufficient to justify the added cost for the health-care system of providing every patient or every general practitioner a PoC device. However, when clinical performance is evaluated including in the outcomes, the increased quality of life for the patient, a tailored and comfortable monitoring procedure, and most importantly the reduced loss in worked (or school) hours, the balance is expected to be in favor of the PoC.

Information required for proper interpretation of TDM measurements for DBS (and other specimens) samples are not different for the ones required for serum sampling. As for traditional serum TDM, the sample (and its results) needs to be accompanied and evaluated with meticulous dosage history, sampling time, and clinical evaluation. Timing of the sampling may be readjusted for saliva sampling according to the drug PK (Jones et al., 2005; Schipper et al., 2007). On one hand, home sampling and sample shipment provide for lower cost and higher portability (in terms of easier management for the patient, who does not have to travel to the larger centers for proper analysis), and less skill for sampling. Yet, the analysis *per se* still requires sophisticated techniques, and is performed in dedicated centers. Generally, the pre-analytical steps for DBS have to be carefully tested for the each specific drug. For example, the hematocrit value is known to affect the viscosity of the blood drop and its deposition on paper (Demirev, 2013; Kong et al., 2014). In the lab, the chemist may choose to evaluate the hematocrit directly, or indirectly via potassium measurements, and proceed to viscosity adjustment before drop deposition. For home sample collection, however, a different protocol has to be established for the DBS sample to be reliable. Most of DBS and saliva methods use mainly chromatographic methods that need to be analytically re-validated (e.g., reference calibration range may need to be reestablished, etc.) and also clinically validated with proper reference concentrations to provide the physician an appropriate interpretation of the data (Ferrante di Ruffano et al., 2012; Halling et al., 2012; Kong et al., 2014). Recently, DBS analyses that do not require complex sample pretreatment have been reported (Espy et al., 2012). Paper-spray based mass (Wang et al., 2010) spectrometry demonstrated capability for whole blood analysis and has been translated in a fully automated sample-to-answer analyzer that will certainly improve the TAT and the analytical TDM pipeline (Manicke et al., 2011), although the instrument is yet too big to fit as a home-based PoC analytical device.

Dried blood spot sample collection is very promising for PoC devices too, as it could naturally find its way into paper-based lateral flow devices that are definitely much more portable. Lateral flow devices are recently gaining increasing and renovated interest. Previously, they were confined to clinically important analyses that require only a yes/no accuracy answer, but no sensitive quantification of the marker. These analyses can be performed with dip test strips and lateral flow tests, such as tests for infection markers for human immunodeficiency virus, hepatitis C virus, hepatitis B (Lee et al., 2010; Gong et al., 2014), tubercolosis (Tsai et al., 2013), malaria (Horning et al., 2014), tests for the presence/absence of multi drug resistance *Staphylococcus aureu*s in pre-surgical screening (Dutta and Dutta, 2006; Yetisen et al., 2013), or the pregnancy test. The latter is another textbook example of lateral flow tests, based on colorimetric detection of the analyte on a paper strip enclosed in a disposable plastic cassette, which provides an internal control, and a simple yes/no answer (Mark et al., 2010).

Lateral flow test (Mark et al., 2010) and more recently paper-based micro analytical devices [usually referred to as μPAD (Lisowski and Zarzycki, 2013; Hu et al., 2014)] are marketed for application in resource-limited settings for their limited costs, portability, and easy disposal management (Fiorini and Chiu, 2005); indeed, much effort is spent in overcoming the difficulties in the development of quantitative lateral flow PoC of comparable (or improved) sensitivity with standard immunoassays (Ge et al., 2012). In view of PoC-derived waste management, paper-based cartridge offer a cheap disposable test strip that is easily disposed of by incineration with reduced hazardous waste management costs.

Technical and analytical challenges for the development of reliable, robust, reproducible paper analytical devices have been extensively reviewed (Sharma et al., 2011; Li et al., 2012; Liana et al., 2012; Yetisen et al., 2013; Hu et al., 2014). Despite the challenges, devices able to treat the blood specimen *in situ* for analysis have been reported, and can provide the basis for advanced DBS handling and analysis. As an example, devices able to perform red blood cell counts and more recently CD4^+^ cell counts (with blood matrix treatment *in situ*) have been marketed to be used as PoC monitoring in HIV-positive patients (Jokerst et al., 2008).

Recently, use of thread as an alternate cheap affordable and robust microfluidic substitute has been reported (Li et al., 2010), although with limited demonstration of quantitative approaches. Paper devices that do not require microfabrication for the fluidic part, but based on their fluidic system on cheaper and promising 3D origami techniques have been presented (Ge et al., 2012). At the moment, quantitative analysis in clinically relevant ranges with paper devices has been demonstrated for monitoring of hepatic enzymes in about 15 min (Pollock et al., 2012), and look a very promising strategy for other commonly assessed biomarkers. As colorimetric readout of results is subjective to variability in the interpretation, use of mobile phones and smartphone is explored both for off-site consultation and as processor devices. Alternatives to read-out technologies that do not exploit electronic devices but are based on counting or signal development time are also under investigation and validation (Roche et al., 2011; Lewis et al., 2013; Lutz et al., 2013) to further expand the reach of diagnostics devices in low-resource settings. Furthermore, much effort is dedicated to the development of embodied miniaturized battery (also in paper) for self-sustained devices (Chen et al., 2014b). All these analytical systems are gaining increasing interest in higher income countries as well, for their speed, user-friendliness, and predicted impact on (cheaper) home-based testing.

To fulfill a long-term view of PoC, many researchers explore alternative matrices to blood in order to avoid even the invasive fingerpricking. Many studies highlighted a common preference for saliva over traditional phlebotomy in a majority of cases among children, parents, and doctors, especially at the onset of the therapy, when the patient is not yet accustomed to needles (Chee et al., 1993; Gorodischer et al., 1997; Tennison et al., 2004). In addition, these less invasive sampling techniques are gaining increasing interests in pharmacological and epidemiological studies as a simple and cost-effective alternative to phlebotomy (Parker and Cubitt, 1999; Ostler et al., 2014). Reproducibility issues of saliva collection in terms of amount of saliva (in stimulated and unstimulated conditions), sampling timing (Wilson, 2005), interference from food and residual drug tablets (Schipper et al., 2007), interference in the MS analysis of the cellulose fibers of some collection devices have been studied and reported (Shirtcliff et al., 2001). Custom-made options (Chee et al., 1993) and many commercial kits are available for sample collection that offer also proprietary buffer for simple viscosity adjustment (Chiappin et al., 2007; Schipper et al., 2007). Albeit not all drugs show correlation between serum and saliva concentrations, saliva is drawing more attention as a suitable specimen as it can be seen as naturally ultrafiltrated plasma (Liu and Delgado, 1999; Za’abi Al et al., 2003; Patsalos and Berry, 2013). Ultrafiltrated plasma sample analyses aim at measuring the free drug concentration, and not the total plasma concentration; it is recommended when some pathological or therapeutic conditions are suspected to alter the bound fraction in blood, therefore modifying the free blood concentration in the patient (Rowland, 1980; Rolan, 1994). Ultrafiltration is a delicate sample pretreatment procedure, and poses analytical challenges to immunoassays, so that most laboratories prefer standard analytical techniques (Rukhadze et al., 2000; Tonic-Ribarska et al., 2012; Patsalos and Berry, 2013). As the amount to be detected is lower in saliva and ultrafiltrated samples compared to plasma specimens, the assay has to be analytically validated with appropriate calibration for lower concentration ranges, which for few drugs happens to be at the sensitivity limit of the assay (Liu and Delgado, 1999; Za’abi Al et al., 2003; Patsalos and Berry, 2013). Saliva measurements are analytically challenging especially for drugs highly bound to plasma proteins (Liu and Delgado, 1999; Za’abi Al et al., 2003; Patsalos and Berry, 2013). Let us take the anticonvulsant tiagabine as an example: total plasma concentration is in the 50–530 nM range; however, of this total amount, 96% is bound to plasma protein, and only 4% is free, meaning that the free drug concentration is in the 2–21 nM range, requiring carefully validated HPLC-UV or HPLC-MS protocols. In addition, even though not commercially available, immunoassay with antibodies in the average micromolar affinity range would perform poorly at so low concentrations (almost three orders of magnitude less of the affinity constant). Carefully designed immunoassays with fluorescence-based detection are reported to efficiently detect concentration in the picomolar range (Liu and Delgado, 1999; Za’abi Al et al., 2003; Patsalos and Berry, 2013). Here, new technological advances like nanomaterial-enhanced miniaturized assays could provide sensitivity improvement at reduced costs with little modification at the implemented TDM pipeline.

## Perspective on Application of Nanotechnology in Developing PoC Devices for Therapeutic Drug Monitoring

Point-of-care devices may be seen as an integrated complex unity composed of the microfluidic and liquid storage for sample handling and refluxing, a sensor component that detects the molecular entities through some receptors; a transducer element that convert the recognition event in a readable signal, for example, an optical signal or an electric current; a controller and finally the “output unit” for result communication. In addition, the sampling unit has to be disposable and contains all the reagents to provide internal control and calibration for every analysis.

Nanotechnology definitively provides the tools for manipulation and characterization of tiny amount of matter at the nanoscale. Although many analytes have serum and saliva concentrations in the easy reachable micromolar range, the small amount of sample available for analysis as a PoC (few microliters of blood, few hundreds of microliters of saliva) calls for techniques able to detect tiny amount of molecules with little preprocessing of the sample, in other word, in a very complex and noisy matrix. For this reason, electrochemical devices with coupled microfluidics able to attract the analyte and reflux the solution over the sensor have been mostly applied (Kusnezow et al., 2006), and sensor geometry can be optimized at the nanoscale for improved sensitivity (Sheehan and Whitman, 2005a; Zheng et al., 2005). Miniaturized fluidic provide nanoliter drop handling and manipulation that further improves microfluidics capabilities already available in the market. Recent advancements in statistical handling of sample distribution like Digital Microfluidic enable nanodrop handling and dispensing with quantitative and sensitive detection of few individual molecules per sample (Nair and Alam, 2008, 2010).

Nanomaterial-enhanced devices may be grouped (for descriptive purposes) into four main categories, depending on the signal detection technology employed. Mechanical sensors have shown great potential in detecting very diluted analytes and few pathological or infective cells (Kelley et al., 2014) by measuring a shift in mechanical properties of the material, like the resonating frequency of the nanostructured sensor. Hybrid nanomechanical and optoplasmonic sensors were recently used to detect cancer biomarker in spiked serum (Bashir, 2004; Arlett et al., 2011) with sub pictogram per liter sensitivity. Similar resonating nanostructured surfaces have been used in controlled environment (i.e., without complex biological matrix) to exploit super-hydrophobic effect to concentrate sparse analyte molecules right at the site of detection (De Angelis et al., 2011; Melli et al., 2011; Dicuangco et al., 2014), although performance in complex biological matrices have not yet been demonstrated in the literature and non-specific binding of abundant serum proteins is a major concern.

Optical read-out technologies span from colorimetric detection (via cameras or by naked eye) to spectrophotometry, fluorescence, turbidimetry, or more sophisticated techniques like frustrated total internal reflection, surface plasmon resonance, and surface enhanced Raman spectroscopy (Luppa et al., 2011). Most of these technologies are not yet amenable for extreme miniaturization; however, examples of small bench-top size instrumentation are already present. The use of enzymatic conversion of chromogenic substrate or nanoparticle-aggregation chromatic change has been used both in homogeneous assay and in lateral flow assay (Luppa et al., 2011) to signal the qualitative and quantitative presence of the target analyte. Electronic based transduction has been very much improved by the use of nanomaterials in sensor surface development, as the detection of the analyte relies upon the change in the electronic property of the sensor surface, and transistor device can be manufactured at low cost. Mainly, these kinds of devices have been proved effective for the detection of nucleic acid sequences, for which the intrinsic high affinity and selectivity of the probing molecules eliminates much of the matrix noises otherwise associated with protein-based detection.

Electrochemical readouts, however, are the most popular transduction in sensors and sensor development as it is much more convenient to measure changes in flow currents through a reporter redox group at the surface sensor. Many nanomaterials, from nanostructured surface, to polymer, metal NP, and carbon nanotubes have shown superior capability as transducer elements. In addition, these systems can be easily coupled to mass-consumer electronic and be implemented in already existing PoC analysis based on the same principle. Carbon nanotubes, for example, effectively provide enhancement of electrodes transduction capabilities, especially coupled with electrochemical detection of the analyte (West and Halas, 2003). Different configurations, designed to be soon compatible with implantable devices, have been published able to recognize and quantify anti-inflammatory drug like naproxene, in spiked serum sample at clinically relevant sensitivity (Popovtzer et al., 2006; Alonso-Lomillo et al., 2010; Pruneanu et al., 2011; Cruz et al., 2014; Ihalainen et al., 2014). This design, for example, is very versatile, as it exploits enzymes involved in hepatic drug metabolism as receptor elements. Although some of these enzyme show higher specificity for certain drugs, their selectivity and specificity in real patient sample (which may present co-medication and interferents) has to be validated.

Nanotechnology offers a plethora of materials that already proved enhanced quantitative sensitivity (Hockstein et al., 2004; Wei et al., 2009; Askim et al., 2013), like nanoparticle-based recognition elements or surface-based nanoelectrodes (Alonso-Lomillo et al., 2009, 2010; Esfandyari-Manesh et al., 2012; Ginja Teixeira et al., 2013). Electronic-polymer-based sensor and nanostructured surface sensors reached clinical trials with nano-electronic “noses” able to “sniff” patterns of volatile organic chemicals that correlates with lung cancer and gastric pathologies (Sheehan and Whitman, 2005b; Squires et al., 2008) and was evaluated in ongoing clinical trials. These systems offer high-sensitivity performance on non-invasive specimen like exhaled breath. In addition, same achievements have been reported previously on the detection via optical readout of toxic gases. A sophisticated statistical analysis (like dedicated variations of principal components analysis) of a dataset of pattern recognition results serve as a “calibration curve” for detection and quantification of the target analyte, that, in this way, can be identified and quantified through an assembled chemiresistor consisting of different, although, cross-reacting elements. This kind of approach is potential interest also for biomarker and therapeutic (and abuse) drug monitoring, thus offering some multiplexing capability and the circumvention of the requirement for highly non-cross-reactive receptor element.

Similar mathematical approaches have been used to detect organic pollutants and drugs via chemometric analysis of the fluorescence or UV spectroscopy of these compounds in waste water and even in plasma and urine (Bruls et al., 2009). However, the need for a spectrophotometer and the limited sensitivity prevents at the moment a thorough exploitation of this method for effective TDM purposes. Environmental sciences studying the effect of drugs and metabolites on waste water treatment and management pushed the development of novel high-sensitivity portable method for monitoring waste water. With some design able to accommodate biological matrices treatment, these promising technologies may be translated into TDM devices. Analytical approaches for environmental studies and quality assessment of the pharmaceutical formulation brought the development of very sensitive electrochemical detection method based on novel nanomaterial-enhanced electrodes with accurate and reliable analysis of drugs in formulations, and in a few cases, in spiked biological matrices (Kelley et al., 2014).

Nanoparticles are employed in a variety of proof-of-principle sensor devices for their size-depend and interfacial properties, that offer intrinsic novel characteristics to the sensors compared to carrier microbeads generally used in current assays. The use of NP builds not only on their properties, but also on the easy of surface modifications for capture molecule immobilization, and for their capability of interrogating the sample themselves compared to the two-dimensional confinement of receptor in surface sensor that may suffer from mass transport and convective flux limitations if not carefully designed (Hill and Mirkin, 2006). Magnetic nanoparticle-based sensors provide enhanced capturing and separation capabilities and are already implemented in clinical practice for intraoperative monitoring (Justino et al., 2013). Interesting hybrid [or “multi-scale” (Agasti et al., 2010)] system have been reported in which both nano and micro materials are exploited. For example, in the biobarcode assay, magnetic microparticles functionalized with the capturing antibodies search for the target analytes, then they are easily concentrated and non-specific elements of the biological matrix removed through a magnetic field. The use of DNA-biobarcode NPs as secondary identification elements allow for sensitive analysis of multiple analytes in a complex mixture in a microfluidic setting (Sukhorukov et al., 2007; Appleyard et al., 2011; Carregal-Romero et al., 2013).

Modified metal NP are gaining interest both as active sensing elements in different applications, such as nucleic acid and protein detection (Cho et al., 2012), and as a strategy to enhance, via nanomaterial modification, the analytical performance of sensor devices (He et al., 2005; Patel et al., 2009; Lin et al., 2011; Tagad et al., 2013). NPs provide with high local density of receptor to enhance analyte binding and detection (de Miguel and Sanders, 1998; Snowden and Anslyn, 1999; Wiskur and Anslyn, 2001; Wright et al., 2005; Späth and König, 2010) and can enforce multiplexing capability (Griss et al., 2014). Similar approaches to capture attomolar concentration of methylmercury with gold nanoparticle-enhanced electrodes have recently been shown by Cho et al. (Hong Enriquez et al., 2012) and other groups showed promising results with other toxic cations in solution (Liu et al., 2011; Cho et al., 2012), via electronic or electrochemical read-out technologies.

Most of the concentration ranges of interest for TDM falls in the micromolar (and sometimes nanomolar range). This means that in a 10 μL whole blood drop (an average fingerprick drop size), there are about 10^12^ (1 nM)–10^15^ (1 μM) molecules to be detected, which is not as challenging as other cases (like detecting <10 circulating tumor cell in a 1 mL blood sample against roughly 10^9^ of red blood cells and 10^6^ of white blood cells (Touw et al., 2005; Dasgupta and Wahed, 2014), to give an order of magnitude). The real advantage of nanomaterials in these applications resides in extensive miniaturization for extreme portability and tailored designed of receptive surfaces. Most of the already available assays rely upon colorimetric/chemiluminescent sensing and are mostly based on immunoassays or enzymatic assays with substrate specificity. Many new AEDs are not tested with the immunoassay format because a suitable specific receptor has not been identified (or it is not yet available competitively) and its development is too costly to be appealing for a company without estimation of short-time payback. In the last 15 years, the field of synthetic receptors showed huge development in the *in silico* design of libraries and of synthetic moieties able to specifically and sensitively capture molecules of choices for different fields of application (de Miguel and Sanders, 1998; Snowden and Anslyn, 1999; Wiskur and Anslyn, 2001; Wright et al., 2005; Späth and König, 2010). Successful examples of tailored synthetic receptor are extremely promising. A synthetic peptide receptor showed excellent sensitivity in detecting methotrexate, an anti-cancer drug, in a miniaturized test that could be re-engineered for other pharmacological molecules (Griss et al., 2014), and bioinformatics efforts provides *in silico* screening of peptide with high affinity for selected target molecules (Hong Enriquez et al., 2012). These kinds of compounds may be repurposed for a library of specific anti-AED (and other drug) receptors providing extreme flexibility in the panel of drug that can be monitored with portable techniques. However, nanoparticle-based library may offer additional benefit. The advantage in using monolayer protected gold NPs, coated with self-assembling bifunctional ligands (Liu et al., 2011; Cho et al., 2012), as highly sensitive receptors lies in the ease to adapt the system to different target molecules and therefore to create libraries of receptor NP to be used receptor elements. For example, the design of a panel of robust inorganic materials with different functionalization (such as self-assembled-monolayer-protected metal particles) as a chemiresistors has provided a very versatile sensor elements for complex mixture recognition of both toxic gases (Lim et al., 2009) and exhaled breath (Barash et al., 2009). As the development of receptor libraries and the production in high yield of those molecules present a significant hurdle for industrial translation, there is much interest in developing combinatorial panels like the aforementioned NP libraries. Because of their robust processability, they are readily adapted to automated synthesis and suitable for high-throughout screening of affinity for target drugs and that offer easy translation of the same surface design to cheaper core material.

It is expected that the introduction of a host of structural and chemical functionalities onto the same nanoscale architecture will enable more accurate, sensitive, and precise sensor systems and will provide more robust platforms for quick, reliable, and portable drug detection as compared to the techniques used now. The combination of novel statistical analysis (like PCA) already brought forward how pattern recognition designs may circumvent the need of highly selective and specific receptor and still provide high-performance reliable analysis of complex matrices. Even the development of nanomaterial-based detector system in which the nanomaterials allow a simple readout in disposable miniaturized systems enable a timely, detailed, and patient-friendly tight monitoring of therapy that will ultimately benefit the patients and improve their quality of life by providing a fully optimized therapeutic regimen.

## Conflict of Interest Statement

The authors declare that the research was conducted in the absence of any commercial or financial relationships that could be construed as a potential conflict of interest.
